# 2.8 μm emission and OH quenching analysis in Ho^3+^ doped fluorotellurite-germanate glasses sensitized by Yb^3+^ and Er^3+^

**DOI:** 10.1038/s41598-017-16937-7

**Published:** 2017-12-01

**Authors:** Junjie Zhang, Yu Lu, Muzhi Cai, Ying Tian, Feifei Huang, Yanyan Guo, Shiqing Xu

**Affiliations:** 1 College of Materials Science and Engineering, China Jiliang University, Hangzhou, 310018 P.R. China; 2 Laboratory of Glasses and Ceramics, Institute of Chemical Science UMR CNRS 6226, University of Rennes 1, 35042 Rennes, France; 3 Collage of Materials Science and Engineering, Changchun University of Science and Technology, Changchun, 130022 P.R. China

## Abstract

The use of Yb^3+^ and Er^3+^ co-doping with Ho^3+^ to enhance and broaden the Ho^3+^: ^5^I_6_ → ^5^I_7_ ~2.8 μm emissions are investigated in the fluorotellurite-germanate glasses. An intense ~3 μm emission with a full width at half maximum (FWHM) of 245 nm is achieved in the Er^3+^/Ho^3+^/Yb^3+^ triply-doped fluorotellurite-germanate glass upon excitation at 980 nm. The glass not only possesses considerably low OH^−^ absorption coefficient (0.189 cm^−1^), but also exhibits low phonon energy (704 cm^−1^). Moreover, the measured lifetime of Ho^3+^: ^5^I_6_ level is as high as 0.218 ms. In addition, the energy transfer rate to hydroxyl groups and quantum efficiency (*η*) of ^5^I_6_ level were calculated in detail by fitting the variations of lifetimes vs. the OH^−^ concentrations. The formation ability and thermal stability of glasses have been improved by introducing GeO_2_ into fluorotellurite glasses. Results reveal that Er^3+^/Ho^3+^/Yb^3+^ triply-doped fluorotellurite-germanate glass is a potential kind of laser glass for efficient 3 μm laser.

## Introduction

With the rapid development of fiber technology and commercial semiconductor lasers in the past decades, mid-infrared (MIR) solid-state lasers have aroused intense interest for their potential applications in minimally invasive surgery, atmospheric monitoring, remote sensing, and scientific research^1–3^. Specially, wideband gain spectra in the 3 μm wavelength region have a significant impact in many different fields of science and technology.

Usually, crystals doped with rare-earth (RE) ions were fabricated and utilized in solid-state lasers to generate coherent emissions at 3 μm, such as Er^3+^ doped YAG^4,5^, GGG^4^, YSGG^4^ and Ho^3+^/Yb^3+^: YSGG^6^. In 1994, Er^3+^: YAG laser, the most intensively studied of the garnet hosts, was commercially available as hermetic 2.94 μm laser modules based on end-pump monolithic design, but the average output power was only 1 W with a slope efficient of 36%^4^. In 2014, under excitation by 975 nm laser diode (LD) arrays, the pulsed 2.94 μm laser of an Er^3+^-doped YAG crystal has reached 30 W average output power and 150 mJ pulse energy^7^. However, due to the high quantum defect, reaching 70%, and significant heat deposition associated laser power scaling, the optimal beam quality factor of M^2^ reach only 12^7^, which indicates the beam quality is far from nearly diffraction limited level. Unlike crystals, fiber lasers are less susceptible to beam quality deterioration by heat deposition, and easier to scale to a moderate average power of 3 μm. Moreover, RE doped glasses are able to be drawn into single mode fibers, which are the most flexible and compact gain media for high efficiency and excellent beam-quality laser generation^8^. Besides, laser glasses not only have broad absorption spectra that relieve the tolerance for the pump sources, but also broad emission spectral regions, which are essential conditions for wavelengths tuning and ultrashort pulse generation.

So far, the most developed mid-infrared 3 μm fiber lasers are based on the RE doped fluoride glass. The higher output power of 24 W was obtained from Er-doped ZBLAN fiber laser by applying an efficient cooling with a combination of fluid cooling over the entire length of the fiber and conductive cooling at both end-faces of the fiber in 2009^9^. A 2938 nm erbium-doped fluoride glass fiber laser delivering a record output power of 30.5 W in continuous wave operation was reported in 2015^10^. Although laser oscillation at wavelength as long as 3.9 μm and ultra-broadband supercontinuum spectra from deep-ultraviolet to MIR have been successfully demonstrated in fluoride glasses, they are still not been widely accepted by the industry due to their relatively inferior stability and fragility^11,12^. Chalcogenide glass is another well-known infrared transmitting material, which exhibits favorable properties for RE doped fiber lasing such as high refractive index resulting in large absorption and emission cross-sections, and generally low phonon energy for efficient radiative processes. Significant efforts have been made to develop the RE doped chalcogenide glass, but it is difficult to draw into fiber due to its relatively low recrystallisation temperature which is close to the fiber drawing temperature^13^. In such case, recently researchers pay more attention to the multicomponent oxide, oxyfluoride glasses or glass ceramics as MIR host materials^14–17^. As an alternative, tellurite glass has attracted a great deal of interest not only for its relatively better chemical, mechanical stability and higher refractive index, but also for lower maximum phonon energy (~700 cm^−1^) among all the oxide glasses, which is helpful to reduce the multi-phonon relaxation rate and favorable for 3 μm emission^18^. Moreover, lasers operating at 1.0, 1.5, and 2.0 μm based on the tellurite fibers have been realized in the past decades^19–21^. Therefore, it is extremely essential to extend the working range further into the longer wavelength region in this promising glass host. However, one notable factor is that tellurite glass has lower glass transition temperature (~350 °C) and poor thermal stability to resist thermal damage at high pumping power. In this work, 10 mol% GeO_2_ is added to fluorotellurite glass to improve thermal stability against crystallization and enhance glass transition temperature against thermal damage at high pumping power. Thus, these features render fluorotellurite-germanate glass as an ideal host for mid-infrared laser material.

Among various rare earth ions, Er^3+^ and Ho^3+^ doped fibers are the most universal way to obtain 3 micron laser output. Compared with Er^3+^: 2.7 μm laser, Ho^3+^ doped fiber laser can achieve longer laser wavelength compared to erbium owing to the smaller energy gap of Ho^3+^: ^5^I_6_ → ^5^I_7_ than that of Er^3+^: ^4^I_11/2_ → ^4^I_13/2_. Moreover, Ho^3+^: 2.8 μm laser overlaps better with the fundamental vibration (3400 cm^−1^) of O-H bonds and therefore presents more precise ablation of shallow tissue layers^22^. Based on this theory, we are working on Ho^3+^ doped glass for 3 μm fluorescence. However, the ~3 μm laser operation cannot be obtained efficiently due to (i) the lack of commercialized laser diodes corresponding to the intrinsic absorption of Ho^3+^ ions, and (ii) the population bottleneck effect that occurs with the ^5^I_6_ → ^5^I_7_ transition which is a self-terminated transition. In order to conquer these problems to turn on the probability to acquire ~3 μm lasing from Ho^3+^, we need (i) a proper sensitizer ion with large absorption cross section for Ho^3+^ ion, and (ii) an appropriate deactivated ion with efficient depopulation of Ho^3+^: ^5^I_7_ for population inversion. Fortunately, Yb^3+^ or Er^3+^ ions can be codoped to improve the absorption band of Ho^3+^ ions at 980 nm. In particular, due to the large absorption and emission cross-section, relatively long lifetime, and simply energy level scheme of Yb^3+^, Ho^3+^/Yb^3+^ codoped ways are recognized to obtain efficient and strong mid-infrared luminescence. So far, 3 μm fluorescence in Ho^3+^/Yb^3+^ codoped glasses has been investigated by researchers^22–24^. In addition, compared with Yb^3+^ ions, Er^3+^ can be used as an intermediate medium to improve indirectly energy transfer efficiency from Yb^3+^ to Er^3+^ then to Ho^3+^, and also transfer its energy to Ho^3+^ ion solely pumped by 980 nm LD^25^. Hence, it can be expected that mid-infrared 3 μm fluorescence can be obtained from the Er^3+^/Ho^3+^/Yb^3+^ triply-doped sample pumped by 980 nm excitation and there is a rare investigation focused on the 3 μm emission from the Er^3+^/Ho^3+^/Yb^3+^ triply-doped glass pumped by 980 nm excitation.

In the present work, we report broadband 3 μm luminescence from Er^3+^/Ho^3+^, Ho^3+^/Yb^3+^ codoped and Er^3+^/Ho^3+^/Yb^3+^ triply-doped fluorotellurite-germanate glasses under a 980 nm LD pump. Efficient 3 μm emissions and lifetimes of ^5^I_6_ level were obtained due to its relative lower phonon energy and hydroxyl content. The energy transfer processes between Er^3+^, Ho^3+^ and Yb^3+^ were fully discussed. Moreover, the lifetime quenching mechanism and quantum efficiency (*η*) in ^5^I_6_ level of Ho^3+^ ion were also presented and analyzed by fitting the variations of lifetimes vs. the OH^−^ concentrations. In addition, the glass formation ability and thermal stability of glasses were studied after introducing GeO_2_ into fluorotellurite glasses. The present work is important to explore the feasibility of implementing mid-infrared lasers with fluorotellurite-germanate glass.

## Experimental

In the Er^3+^/Ho^3+^/Yb^3+^ triply-doped system, if the concentration of Yb^3+^ and Er^3+^ are too small, the absorption coefficient at around 980 nm would be greatly reduced, resulting in a lower pumping absorption efficiency. On the contrary, if the concentration of Yb^3+^ and Er^3+^ are too large, the Yb^3+^ or Er^3+^ ion would form cluster structures involving at least Yb^3+^-Yb^3+^ or Er^3+^-Er^3+^ ions pairs in fluorotellurite-germanate glass when the Yb^3+^ and Er^3+^ doped concentration were larger than 2 mol% and 0.5 mol% respectively, which would result in the fluorescence quenching^26,27^. A middle-ground approach was taken in our experiment, the concentration ratio between Ho^3+^, Er^3+^and Yb^3+^ was chose as 1:1:4, they were 0.5, 0.5 and 2 mol%, respectively.

Glasses were developed with molar composition of (75-x)TeO_2_-10Nb_2_O_5_-12YF_3_ -xGeO_2_-0.5HoF_3_-0.5ErF_3_-2YbF_3_ (x = 0, 10) denoted as T1 and TG1, respectively. At the same time, 0.5Ho^3+^/0.5Er^3+^ and 0.5Ho^3+^/2Yb^3+^ codoped fluorotellurite (T) and fluorotellurite-germanate (TG) samples were also prepared to make a comparison and denoted as T2, T3, TG2 and TG3, respectively. The glasses were prepared by the conventional melting-quenching technique, using high-purity TeO_2_ (99.99%), GeO_2_ (99.99%), Nb_2_O_5_, YF_3_, HoF_3_ (99.99%), ErF_3_ (99.99%) and YbF_3_ (99.99%) powders. Well-mixed 15 g batches were melted at 900 °C for 18 min in a platinum crucible. Then the melts are poured into reheated molds and annealed for 2 h near the glass transition temperature before they are cooled to room temperature. Other glass samples with the same components of T1, T2, T3, TG1, TG2 and TG3, named T1O, T2O, T3O, TG1O, TG2O and TG3O, were prepared using the same process described above, except that the batch of T1O, T2O, T3O, TG1O, TG2O and TG3O were first dried in a vacuum drying oven at 100 °C for 24 h before melting to remove the crystal water content in the raw materials, and dried O_2_ was shielded into the glass melt for 15 minutes to eliminate OH^−^. Finally, the annealed samples are fabricated and polished to the size of 10 × 10 × 1.5 mm^3^ for the optical and spectroscopic measurements, while others are cut and polished for refractive index.

The refractive index and density of the glasses were measured by the prism minimum deviation and Archimedes methods using distilled water as the immersion liquid. Differential scanning calorimeter (DSC) curve is measured using NETZSCH DTA 404 PC at the heating rate of 10 K/min with maximum error of ±5 °C. The Raman spectrometer (Bruker, Switzerland) was used with a 532 nm laser as the excitation source. The fluorescence spectra in the range of 2500~3100 nm were measured by using a steady state spectrometer (FLSP 980) (Edingburg Co., England) pumped at 980 nm LD with the output power of 600 mW. The decay curves at 2.8 μm fluorescence were obtained with light pulses of the 980 nm LD with the same power and HP546800B 100-MHz oscilloscope. The infrared transmittance spectra were obtained with a Thermo Nicolet (FTIR spectrometer) spectrophotometer in a region between 2.5 and 4.0 μm, with a resolution of 4 cm^−1^. To get comparable results, same excitation power and distance between the sample and pumping source were maintained when different samples were taken the mid-infrared, visible emission spectra and lifetime measurements. All the measurements were carried out at room temperature.

## Results

### Thermal, mechanical stability and structure analysis

**Figure 1:**
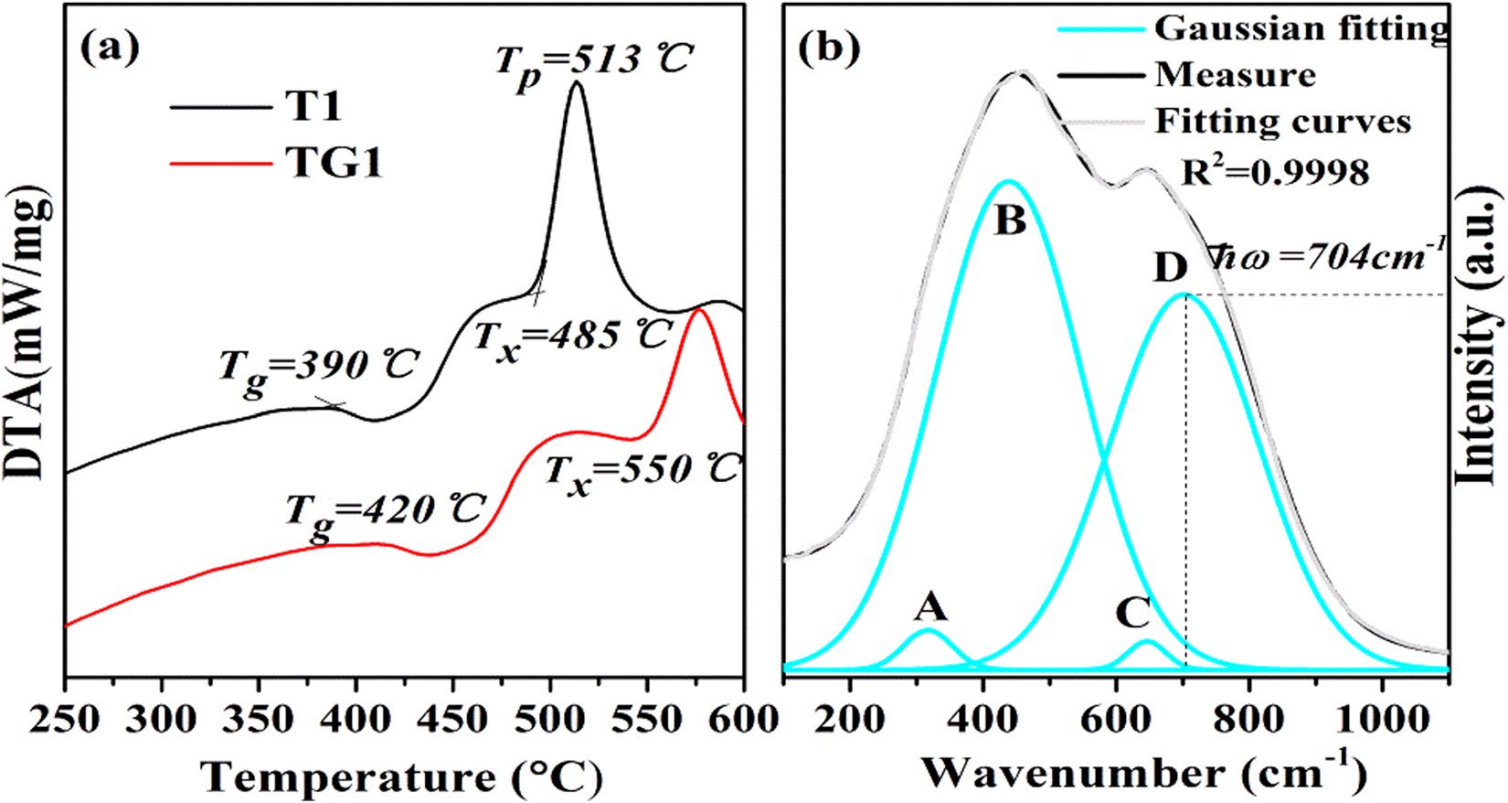
(**a**) DSC curves of the T1 and TG1 glasses. (**b**) Raman spectrum of the TG1 glass with fitting data.

Thermal stability is one of the most important properties for glass and fiber drawing, which determines whether the working temperature range of fiber drawing is wide enough. Since the fiber drawing is a reheating process, any crystallization or phase separation will ultimately increase the optical loss and worsen the transmission characteristics of the fiber. Generally, four technological parameters including glass transition temperature (T_g_), onset crystallization temperature (T_x_), peak crystallization temperature (T_p_) and their temperature difference (ΔT = T_x_ − T_g_) are frequently used to evaluate the glass thermal properties. The first three temperature parameters are determined from the tangent intersections of DSC curves. A larger ΔT, especially much larger than 100 °C, indicates the glass possesses an excellent thermal ability against the nucleation and crystallization^28^. Figure 1(a) displays the DSC curve of fluorotellurite and fluorotellurite-germanate glasses (T1 and TG1). It is found that the ΔT of TG1 sample is 130 °C, which is significantly larger than that of T1 sample (95 °C). It is also higher than that of TeO_2_-ZnO-Na_2_O glass system (114 °C)^29^, fluoride glass (85 °C)^30^, germanate-tellurite (122 °C)^31^ and lower that of germanate glass (190 °C)^32^, which reveals that introducing GeO_2_ into fluorotellurite glasses can improve a wide operating temperature range and glass stability against crystal nucleation and growth during the fiber drawing process. Furthermore, T_g_ is an important factor for laser glass, the one (420 °C) of TG1 sample is higher than that of T1 glass (390 °C), TeO_2_-ZnO-Na_2_O glass system (303 °C)^29^, germanate-tellurite (398 °C)^31^, compared to fluoride (427 °C)^30^, but lower than that of germanate (660 °C)^32^, which indicate that this fluorotellurite-germanate glass has better resistance to the thermal damage aroused by the transmitted high-power laser, namely, higher laser-induced damage threshold.

To contrast and estimate more comprehensively the thermal stability of developed samples (T1 and TG1), the parameter S is employed and defined by

1 $$S=[({T}_{p}-{T}_{x})({T}_{x}-{T}_{g})]/{T}_{g}$$

where (*T*_*p*_ − *T*_*x*_) is related to the rate of devitrification transformation of the glassy phases. On the other hand, the high value of ΔT delays the nucleation process. It is found that the S of TG1 sample is as high as 8.67 K. It is evidently higher than those of T1 sample, TeO_2_-ZnO-Na_2_O^29^, fluoride^32^, germanate^30^ glasses and compared to that of germanate-tellurite^31^ glass as shown in Table 1. Therefore, the prepared fluorotellurite-germanate glass (TG1) has strong resistance to devitrification after the formation of the glass and might has potential application in fiber laser.

**Table 1:** The glass transition temperature (T_g_), onset crystallization temperature (T_x_), temperature of crystallization peak (T_p_), thermal stability parameters ΔT and S in various glass hosts.

Glass samples	T_g_ (°C)	T_x_ (°C)	T_p_ (°C)	ΔT (°C)	S (K)	References
T1	390	485	513	95	6.82	This work
TG1	420	550	578	130	8.67	
Tellurite	303	417	435	114	6.76	29
Germanate	660	850	875	190	5.09	32
Fluoride	427	512	535	85	4.58	30
Germanate-tellurite	398	472	520	122	8.92	31

The durability in water is an important factor to evaluate the chemically durable properties of fluorotellurite-germanate glass, these properties were measured as follows:

2 $${\rm{W}} \% =\frac{{W}_{1}-{W}_{2}}{{{\rm{W}}}_{{\rm{2}}}}\times 100 \% \quad {\rm{W}}^{\prime}  \% \frac{{W}_{1}-{W}_{2}}{{\rm{V}}}\times 100 \% $$

The V and ρ are the volume and density of the sample, respectively. The sample (W_1_) was weighed firstly, then that was weighed again (W_2_) after the glass was then stayed around the constant temperature water bath glass beaker at 98 °C for 24 h to the sample was cooled and dried in a annealing furnace at 70 °C for 1 h.

The chemically durable of present fluorotellurite-germanate glass (TG1O) is evaluated based on the weight loss experiment. The W% (71.5 mg/g) and W′% (249.7 mg/cm^3^) of sample from the above formula (2), are approximately half of that of ZBLAN glass^30^ and also lower than that of fluoro-tellurite^33^, even compared to that of germanate glass^16^.

In order to further quantitatively evaluate the mechanical strength properties of fluorotellurite-germanate glass (TG1), these bending strength (*B*) and compression strength (σ) were measured as follows^34^:

3 $$B=\frac{3{F}_{1}L}{2b{t}^{2}}\quad \quad \sigma =\frac{{F}_{2}}{b{t}^{2}}$$

where the *L*, *b* and *t* is length of sample, width of sample and thickness of sample, respectively. *F*_*1*_ is yield stress, which was measured with the WDW-2E universal testing machine. *F*_*2*_ is fracture stress, which was measured with the CMT5105 electromechanical universal testing machine.

The *B* (32.66 MPa) and σ (135.12 MPa) of sample from the above formula (3), are compared to those of SiO_2_-Al_2_O_3_-MgO glass system^34^. Thus, these features render fluorotellurite-germanate glass as an ideal host for mid-infrared laser material.

Raman spectrum is an effective way to study the structure of glass materials. Figure 1(b) shows the measured Raman spectra of the fluorotellurite-germanate (TG1) glass with fitting data in the spectral range of 100~1100 cm^−1^. There mainly exist two broad continuous scattering peaks attributed to the disordered structures in the present glass. The spectra can be further decomposed into four symmetrical Gaussian peaks (denoted as A, B, C and D), including two medium peaks around 314 and 704 cm^−1^, and two strong peaks around 442 and 644 cm^−1^. These fitted peak positions are derived from the data reported for other similar fluorotellurite-germanate glasses^31,35^. All of these peaks are ascribed to the vibrations of the coordination polyhedral tellurium and germanium. The peaks around 462 and 670 cm^−1^ can be assigned to the asymmetric stretching vibrations of Ge-O-Ge and Te-O-Te linkages formed by sharing vertices of the TeO_4_ trigonal bipyramid (tbp) units, TeO_3+1_ polyhedra or TeO_3_ trigonal pyramid (tp) units. Those around 314 and 704 cm^−1^ may originate from the bending vibrations of Te-O bond and Te = O double bonds in [TeO_3_] and distorted [TeO_3+δ_] trigonal pyramidal. Hence, the presence of multiple structural sites in the present fluorotellurite-germanate glass may be in favor of yielding an inhomogeneously broadened spectrum and improving the solubility of RE ions. In addition, the lower phonon energy could reduce the probability of non-radiative relaxation and thus be helpful to Ho^3+^ 2.8 μm luminescence. It can be found that the maximum phonon energy of the present glass only extends to 704 cm^−1^, which is much lower than that of tungsten tellurite (~920 cm^−1^)^36^, germanate (~845 cm^−1^)^37^ and germanate-tellurite glasses (~764 cm^−1^)^31^. In general, the highest phonon frequencies of the matrix should be around 0.2~0.25 times less than the light frequency in order to emit at long wavelengths^38^. For ~3.0 μm fluorescence, the maximum phonon frequency of the host medium should be smaller than 833 cm^−1^. Therefore, this fluorotellurite-germanate glass with smaller phonon energy (704 cm^−1^) could reduce the non-radiative relaxation probability of Ho^3+^ efficiently and thus be very conducive to Ho^3+^: 2.8 μm luminescence.

### Absorption and infrared transmittance spectrum

Based on previous reports^22,23,25^, the absorption spectrum of Ho^3+^ singly doped sample cannot match well with readily available laser diodes, such as 808 and 980 nm. Fortunately, Er^3+^/Ho^3+^ and Ho^3+^/Yb^3+^ codoped samples display an obvious absorption band around 980 nm owing to the absorption transition of Er^3+^: ^4^I_15/2_ → ^4^I_11/2_ and Yb^3+^: ^2^F_7/2_ → ^2^F_5/2_. Therefore, the prepared Er^3+^/Ho^3+^/Yb^3+^ triply-doped glass can be excited by commercially 980 nm laser diode and better 3 μm spectroscopic properties may be obtained because of double activation effects.

**Figure 2:**
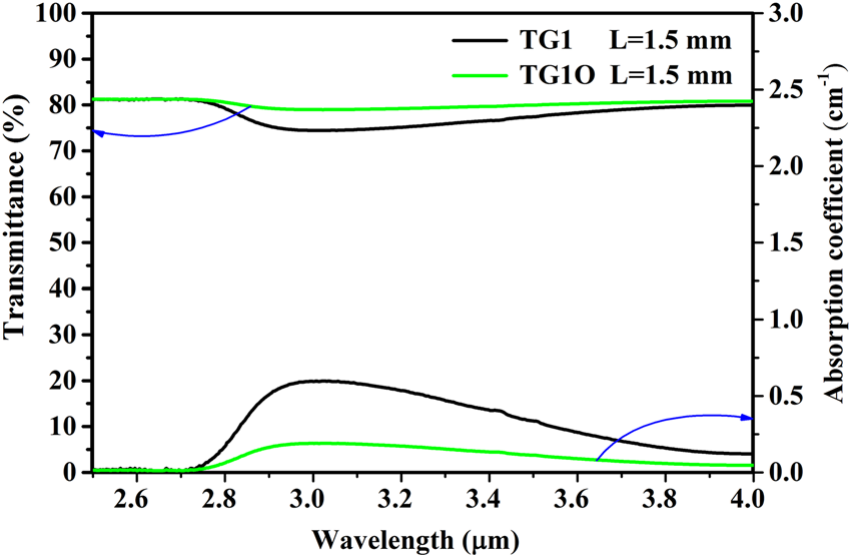
Infrared transmittance spectrum of TG1 and TG1O glasses.

As maximum phonon energy of the present glass, the OH^−^ absorption coefficient at about 3 μm is the key to application of fluorotellurite-germanate glass. The Fig. 2 shows the infrared transmittance spectrum of TG1 and TG1O (with the shielding gas (O_2_)) samples at 1.5 mm thick. The transmittance reaches as high as 81% for TG1 and TG1O at 2.7 μm band under an uncontrolled atmosphere, which is beneficial for 3 μm emission. The residual loss contains the Fresnel reflection, dispersion and absorption of samples. It is noted that the absorption band at 3 μm is ascribed to the vibration of hydroxyl groups. A previous study on tellurite-germanate glasses showed that the absorption bands of OH groups in oxide glasses can be classified into three groups: (1) free OH groups at 3500 cm^−1^, (2) strongly bonded OH groups at 2650 cm^−1^, and (3) very strongly bonded OH groups at 2200 cm^−1^. As shown in Fig. 2, free OH groups play a major role in the IR absorption of the glass. Therefore, the contents of OH^−^ groups have an influence on mid-infrared fluorescence. In addition, it can be seen that the utilization of the shielding gas (O_2_) could bring about a better dehydration result, which can be associated with the depressed incorporation of environmental H_2_O and the facilitated evaporation of OH^−^ from the melt into outside environment. The absorption coefficient *α*_*OH*_ (cm^−1^) in the glass network can be evaluated with the following equation^39^:

4 $${\alpha }_{O{H}^{-}}=\frac{\mathrm{ln}(T/{T}_{0})}{L}$$

where *L* is the thickness of the sample, *T* is the transmission at 3500 cm^−1^, and *T*_0_ is the transmission of the glass matrix. Furthermore, the OH^−^ concentration (*N*_*OH*_) is obtained from the absorption coefficient by Eq. (5)^39^:

5 $${N}_{O{H}^{-}}=\frac{{N}_{A}}{\varepsilon }{\alpha }_{O{H}^{-}}$$

The value ε is the molar absorptivity corresponding to OH^−^ in silicate glasses (49.1 × 10^3^ cm^2^/mol)^36^ and *N*_*A*_ is the Avogadro constant (6.02 × 10^23^/mol). The absorption coefficient (0.189 cm^−1^) and OH^−^ concentration (0.232 × 10^19^ cm^−3^) of the TG1O sample are significantly lower than TG1 sample (0.597 cm^−1^ and 0.729 × 10^19^ cm^−3^), which demonstrated that the simultaneous utilization of shielding gas (O_2_) is an effective method to extract OH^−^ out of the mid-infrared laser glass during the fabrication process. Similarly, all other TG samples and T samples were also tested and summarized in Table 2. The absorption coefficients (*α*_*OH*_) and OH^−^ concentrations (*N*_*OH*_) of the other samples are comparable to those of samples (TG1, TG1O). Besides, the minimal *α*_*OH*_ (0.179 cm^−1^) and *N*_*OH*_ (0.221 × 10^19^ cm^−3^) are much lower in comparison with other tellurite and germanate glasses reported before^22,39^. Hence, the low content of OH groups can make the prepared glass a promising mid-infrared laser material.

**Table 2:** The absorption coefficients *α*_*OH*_ (cm^−1^) and OH^−^ concentrations *N*_*OH*_ (×10^19^ cm^−3^) of all T and TG samples.

Sample	T Glass					
	T1	T1O	T2	T2O	T3	T3O
*α*_*OH*_ *(cm* ^−*1*^)	0.543	0.181	0.549	0.179	0.538	0.187
*N*_*OH*_ (*10 ^*19*^ *cm* ^*−3*^ *)*	0.666	0.223	0.673	0.221	0.660	0.230
**Sample**	**T G Glass**					
	**TG1**	**TG1O**	**TG2**	**TG2O**	**TG3**	**TG3O**
*α*_*OH*_ *(cm* ^*−1*^ *)*	0.597	0.189	0.592	0.184	0.603	0.192
*N*_*OH*_ (*10 ^*19*^ *cm* ^−*3*^)	0.729	0.232	0.726	0.226	0.739	0.235

### Analysis of fluorescence spectra and energy transfer mechanism

**Figure 3:**
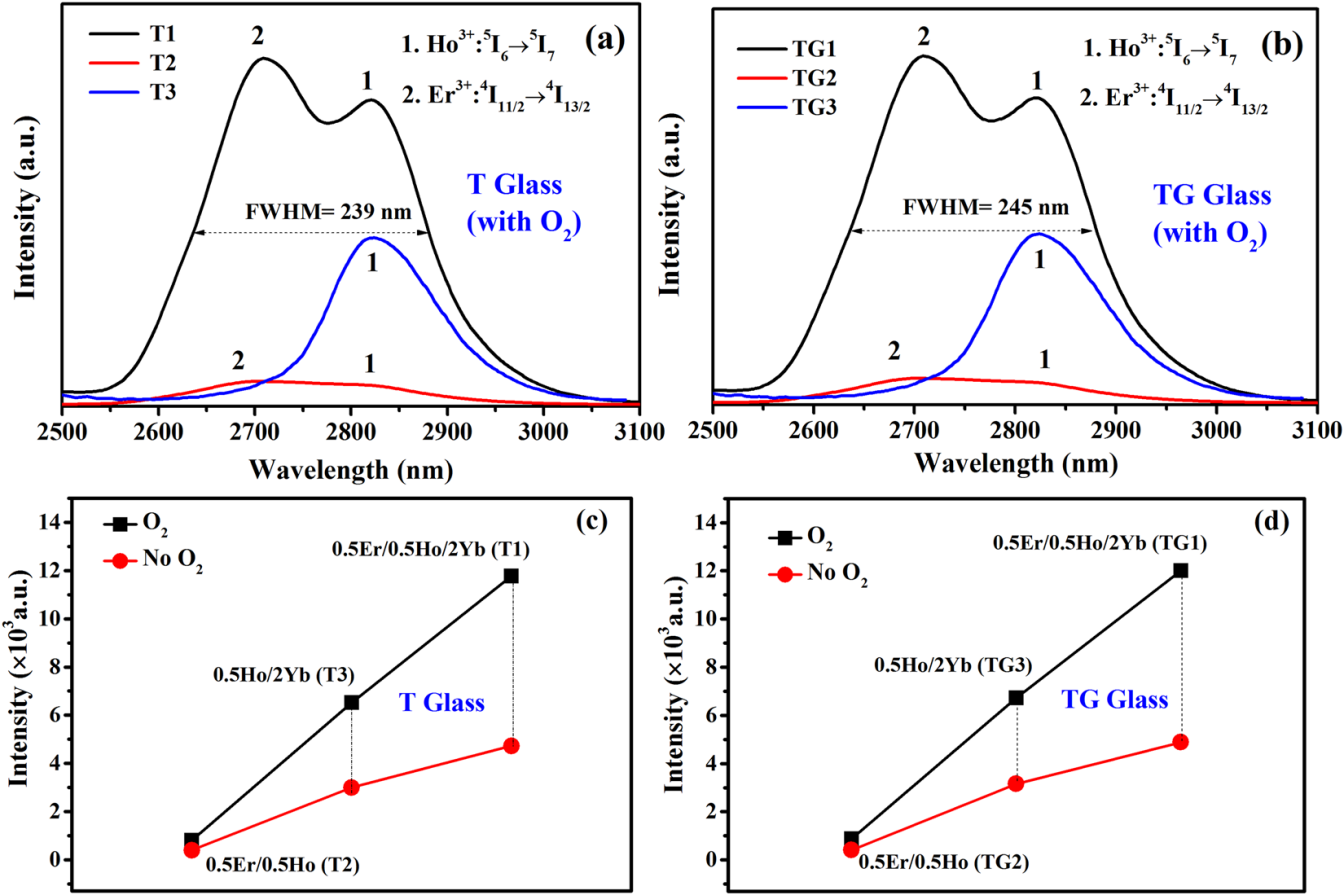
(**a**,**b**) 3 μm fluorescence spectra of T and TG glasses (with the shielding gas (O_2_)) pumped at 980 nm. (**c**,**d**) Ho^3+^: 2.83 μm emission intensity (red squares) of the T and TG samples (without O_2_); (black squares) of the T and TG samples (with O_2_).

Figure 3(a) presents fluorescence spectra of 0.5Er^3+^/0.5Ho^3+^, 0.5Ho^3+^/2Yb^3+^ codoped and 0.5Er^3+^/0.5Ho^3+^/2Yb^3+^ triply-doped fluorotellurite (T) glasses (with the shielding gas (O_2_)) in the region of 2500~3100 nm pumped at 980 nm. All the samples were measured under the same conditions. It is obvious that the 2.83 μm emission is more intense in the triply doped sample than these of other samples, which indicates efficient energy transfer between Yb^3+^, Er^3+^ and Ho^3+^. Moreover, the 2.71 μm emissions of 0.5Er^3+^/0.5Ho^3+^ codoped and 0.5Er^3+^/0.5Ho^3+^/2Yb^3+^ triply-doped samples were observed due to the Er^3+^: ^4^I_11/2_ → ^4^I_13/2_ transition. However, there is no obvious 2.83 μm emission band in the Er^3+^/Ho^3+^ codoped sample, because of the smaller absorption cross-section of Er^3+^ pumped at 980 nm compared to Yb^3+^. The same situation also appears in fluorotellurite-germanate (TG) glass (with the shielding gas (O_2_)) as shown in Fig. 3(b). In this research, from the Fig. 3(c) and (d), it can be seen that Ho^3+^: 2.83 μm emission intensity of Ho^3+^/Yb^3+^/Er^3+^ and Ho^3+^/Yb^3+^ codoped T and TG glasses are also quite strong. This same phenomenon also appears in the T and TG samples (without the shielding gas (O_2_)). It proves that Er^3+^/Ho^3+^/Yb^3+^ and Ho^3+^/Yb^3+^ codoped samples are both suitable sensitizing methods to achieve strong 3 μm emission by pumping at 980 nm, but the triply doped sample is even better. In addition, the 2.83 μm emission intensity of 0.5Er^3+^/0.5Ho^3+^/2Yb^3+^ triply-doped TG glass is slightly higher than that of T glass, which suggests that Er^3+^/Ho^3+^/Yb^3+^ triply doped TG glass can be more alternative way to get 3 μm emission. Moreover, a flat ultra-wideband emission from about 2500 to 3100 nm with a maximum full width at half maximum (FWHM) of 245 nm is obtained from the Er^3+^/Ho^3+^/Yb^3+^ triply doped TG sample. Therefore, the Er^3+^/Ho^3+^/Yb^3+^ triply doped TG glass with ultra-wideband emission has potential application in mid-infrared fiber amplifier and broad band tunable lasers.

**Figure 4:**
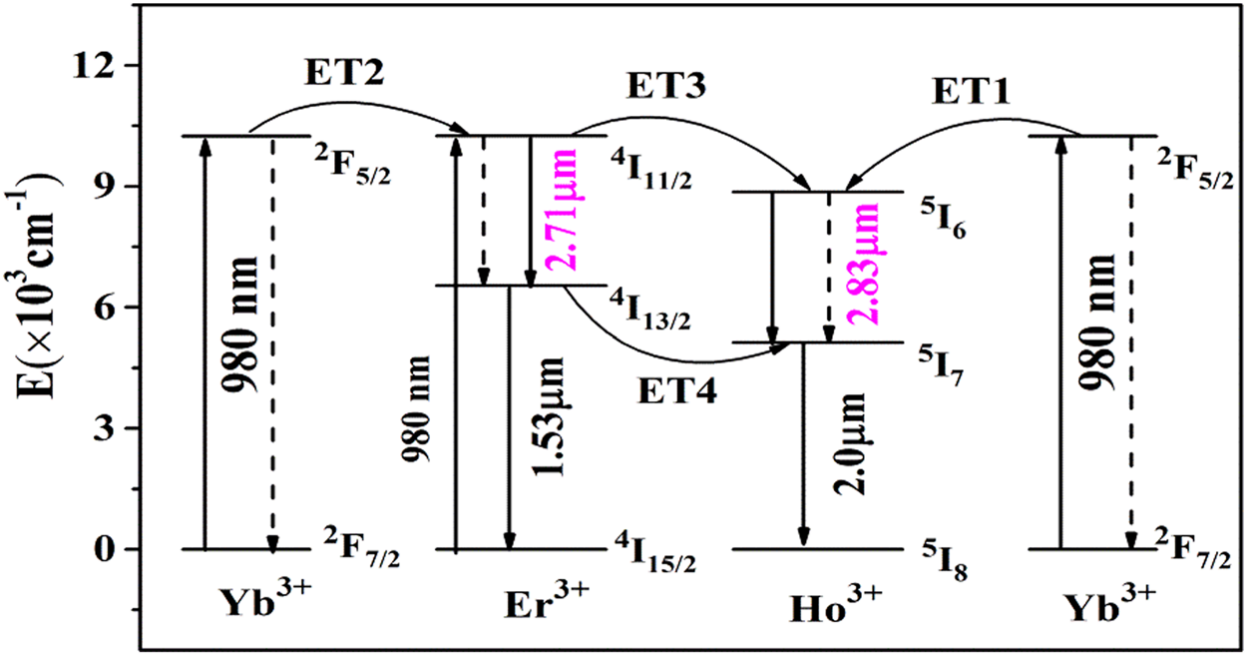
Energy level schemes and energy transfer processes between Ho^3+^, Er^3+^ and Yb^3+^.

In order to explain the above emission spectra, the schematic diagram of level transition of Yb^3+^-Er^3+^-Ho^3+^ ions is presented in Fig. 4. The ions in the Yb^3+^: ^2^F_7/2_ levels are pumped to a higher ^2^F_5/2_ level via ground state absorption (GSA: Yb^3+^: ^2^F_7/2_ + a photon → ^2^F_5/2_). Similarly, the ions in the Er^3+^: ^4^I_15/2_ level are also pumped to a higher ^4^I_11/2_ level via ground state absorption (GSA: Er^3+^: ^4^I_15/2_ + a photon → ^4^I_11/2_) when excited by commercial 980 nm LD. The ^2^F_5/2_ level transfers a part of its energy to the adjacent Ho^3+^: ^5^I_6_ level (ET1: Yb^3+^: ^2^F_5/2_ + Ho^3+^: ^5^I_8_ → Yb^3+^: ^2^F_7/2_ + Ho^3+^: ^5^I_6_), and Er^3+^: ^4^I_11/2_ level (ET2: Yb^3+^: ^2^F_5/2_ + Er^3+^: ^4^I_15/2_ → Yb^3+^: ^2^F_7/2_ + Er^3+^: ^4^I_11/2_), making their energy levels populated. On the one hand, the ions in the Er^3+^: ^4^I_11/2_ level can relax to the lower ^4^I_13/2_ level by a nonradiative process and radiative relaxation (2.71 μm emission). Then, the ^4^I_13/2_ level transfers a part of its energy to the adjacent Ho^3+^: ^5^I_7_ level (ET4: Er^3+^: ^4^I_13/2_ + Ho^3+^: ^5^I_8_ → Er^3+^: ^4^I_15/2_ + Ho^3+^: ^5^I_7_), making this energy level populated. In addition, some ions in the ^4^I_13/2_ level radiate to the ground state (^4^I_15/2_), resulting in 1.53 μm emissions (Er^3+^: ^4^I_13/2_ → ^4^I_15/2_ + 1.53 μm). On the other hand, the Er^3+^: ^4^I_11/2_ level can also transfer its energy to the Ho^3+^: ^5^I_6_ level via an ET3 (Er^3+^: ^4^I_11/2_ + Ho^3+^: ^5^I_8_ → Er^3+^: ^4^I_15/2_ + Ho^3+^: ^5^I_6_) process. Finally, 2.83 μm emission takes place due to radiative transition to the state (^5^I_7_ level) from Ho^3+^: ^5^I_6_ level (Ho^3+^: ^5^I_6_ → ^5^I_7_ + 2.83 μm). In addition, some ions in the ^5^I_7_ level radiate to the ground state (^5^I_8_), resulting in 2.0 μm emissions (Ho^3+^: ^5^I_7_ → ^5^I_8_ + 2.0 μm). Basing on discussions mentioned above we can summarize that both ET1-3 processes can generate 2.8 μm fluorescence, but ET4 process is not beneficial for 2.8 μm fluorescence. However, from Fig. 3(a), it is found that the positive effect of the energy transfer process (ET3) is greater than the negative effects of energy transfer process (ET4), so the introduction of Er, as Ho ion is favorable for Ho^3+^ 2.8 μm fluorescence.

### Analysis of 2.83 μm lifetime and energy transfer to OH^−^ groups

**Figure 5:**
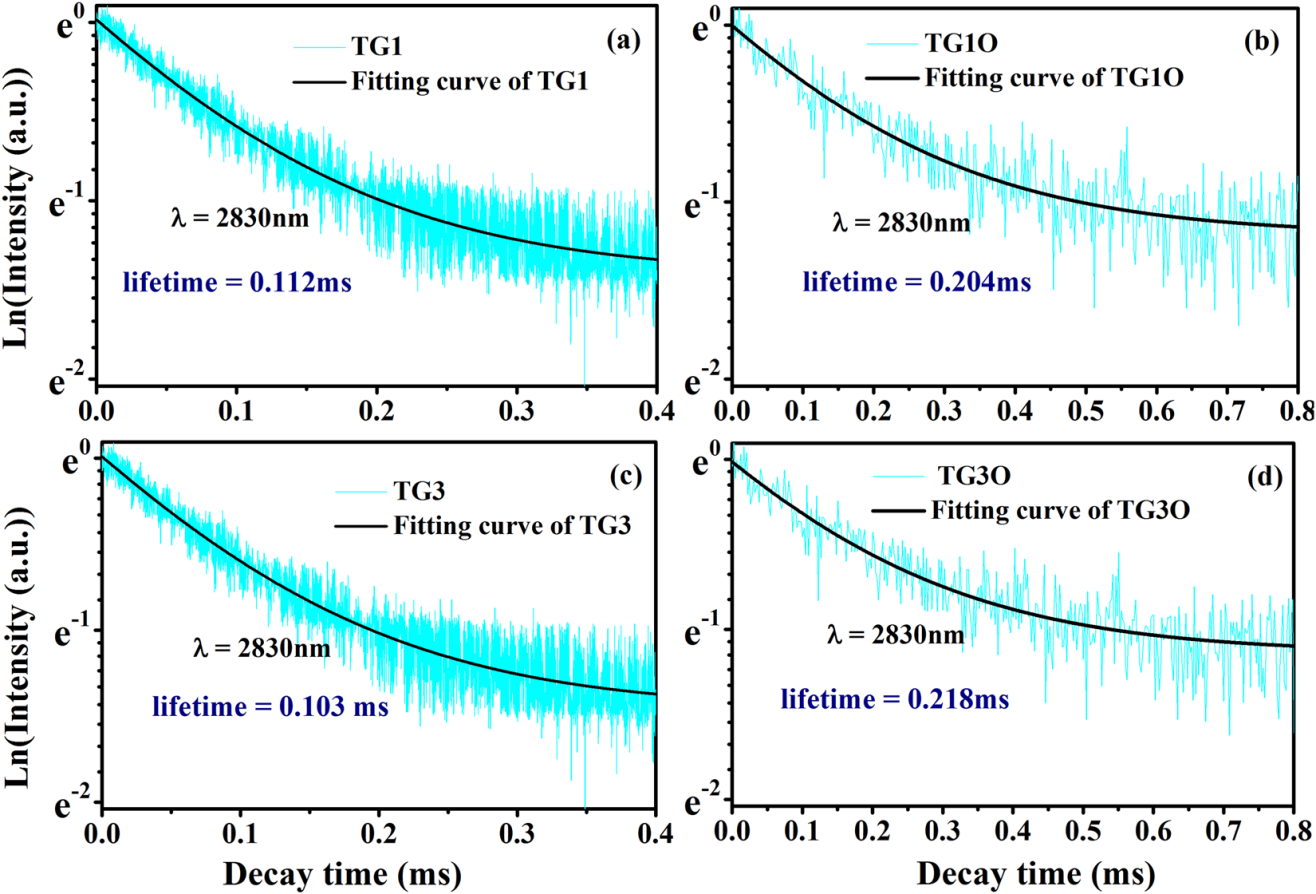
(**a**–**d**) Fluorescence decay curves of the Ho^3+^: ^5^I_6_ energy level of 0.5Ho^3+^/2Yb^3+^, 0.5Er^3+^/0.5Ho^3+^/2Yb^3+^ doped fluorotellurite-germanate glasses.

A long fluorescence lifetime is another important factor in the success of Ho^3+^ doped fiber laser. Even though Ho^3+^ ions have been widely doped into different host materials, the measured lifetime τ at ^5^I_6_ level was rarely reported in germanate or tellurite glasses, which may be due to their extremely weak emission intensity beyond the detection range of current facilities. However, the decay curves of the ^5^I_6_ level of Ho^3+^ doped fluorotellurite-germanate glasses are measured by light pulse of the 980 nm LD with producing a pulse with a width of 50 μs and a repetition rate of 10 Hz in the an HP546800B 100-MHz oscilloscope. The experimental lifetimes are determined by the procedure of single exponential fitting. The measured decay curves of 0.5Ho^3+^/2Yb^3+^ (TG3 and TG3O) codoped, 0.5Er^3+^/0.5Ho^3+^/2Yb^3+^ (TG1 and TG1O) triply doped samples and the fitted lifetimes are showed in Fig. 5. Here, we did not acquire accurate decay curves for the 0.5Er^3+^/0.5Ho^3+^ (TG2 and TG2O) codoped samples because of the weak fluorescence intensity under short pulse pumping condition. Figure 5 shows that the fluorescence decay characteristic at 2.83 μm and the measured lifetimes τ of TG3O and TG1O were estimated to be 0.204, 0.218 ms, respectively. The measured lifetimes are observed in the fluorotellurite-germanate glasses, and also much larger than that of Y_3_Al_5_O_12_ crystal (0.045 ms)^40^. Thus, this kind of Ho^3+^-excited fluorotellurite-germanate glass has potential application in mid-infrared fiber lasers.

**Figure 6:**
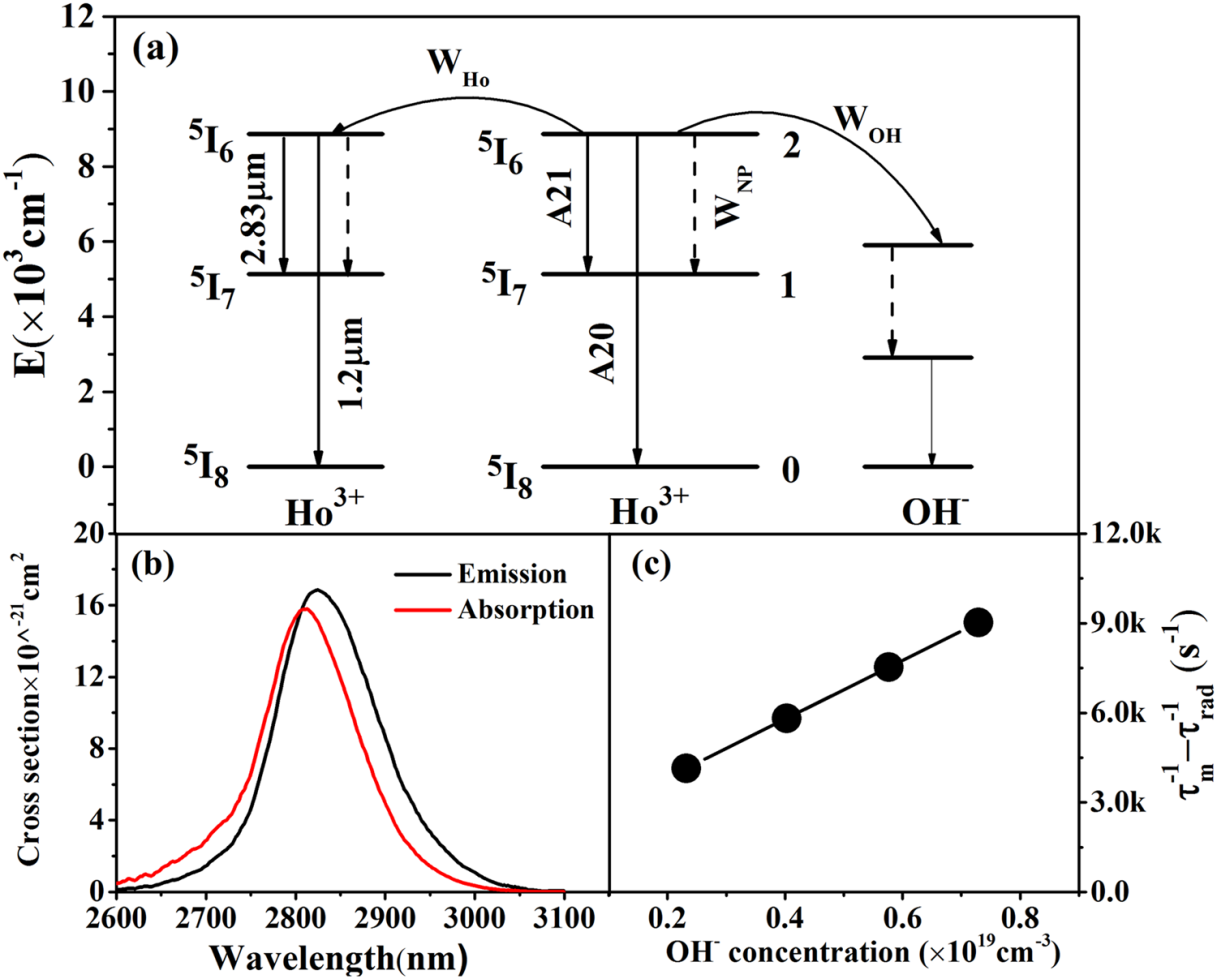
(**a**) Radiation transition and nonradiation transition processes of Ho^3+^ in the fluorotellurite-germanate glass; (**b**) Overlaps of absorption and emission cross-section spectra of the 2.83 μm band; (**c**) *τ*_*m*_^−1^ – *τ*_*rad*_^−1^ as a function of OH^−^ concentration.

It is worth mentioning that the 2.83 μm fluorescence lifetimes of ^5^I_6_ → ^5^I_7_ transition of TG3 and TG1 samples are 0.112 and 0.103 ms respectively, which are half of TG3O and TG1O samples. It can be attributed to the utilization of the shielding gas (O_2_) in the process of preparing glass, which could bring about a better dehydration result and enhance the 2.83 μm fluorescence and lifetimes. In addition, the fluorescence decay curves are strait lines respect to a log scale of Y-axis, which indicate that there is no other significant nonlinear energy transfer between Ho^3+^ ions involved. Based on the previous analysis^41^, a possible mechanism for the lifetime decreasing may be the energy transfer to the OH^−^ groups^42^. Figure 6(a) presents radiation transition process and nonradiation transition process of Ho^3+^ in the fluorotellurite-germanate glass. Here, taking the TG3 and TG3O samples as examples, other nonradiation transition processes (multiphonon decay and energy transfer rate between Ho^3+^ ions) need to be considered. Therefore, in order to clearly elucidate and evaluate the energy transfer rate between Ho^3+^ and OH^−^ groups, and quantum efficiency of Ho^3+^ ions, the measure lifetime *τ*_*rad*_ of Ho^3+^-excited state is finally given by^42^

6 $${\tau }_{m}^{-1}={\tau }_{rad}^{-1}+{W}_{MP}+{W}_{OH}+{W}_{Ho}$$

where *W*_*MP*_ is the multiphonon decay rate from ^5^I_6_ to ^5^I_7_ level taken as a constant and *W*_*Ho*_ is the energy transfer rate between Ho^3+^ ions as also a constant here, *W*_*OH*_ is the energy transfer rate between Ho^3+^ and OH^−^ groups. Here, *W*_*OH*_ can be expressed as^43^

7 $${W}_{OH}=\frac{9}{2\pi }\frac{{N}_{H{o}_{{3}^{+}}}(\omega {N}_{OH})}{{\tau }_{rad}{N}_{0}^{2}}$$

where *N*_*OH*_ and *N*_*Ho*_^*3*+^ are the concentrations of OH^−^ groups and Ho^3+^ ions, respectively. The different OH concentrations were obtained by shielding dried O_2_ into the glass melt for 5, 10 and 15 minutes to eliminate OH^−^, respectively. *ω* represents the proportion of Ho^3+^ ions coupled to OH^−^ groups. *N*_0_ is the critical concentration defined as

8 $${N}_{0}={(4\pi {R}_{0}^{3}/3)}^{-1}$$

where *R*_0_ is the critical distance at which the energy transfer for an isolated donor-acceptor pair separated by *R*_0_ occurs with the same rate as the spontaneous deactivation in the donor itself. *R*_0_ is given by^39^

9 $${R}_{0}^{6}=\frac{3c{\tau }_{rad}}{8{\pi }^{4}{n}^{2}}\frac{{g}_{low}^{D}}{{g}_{up}^{D}}\int {\sigma }_{em}^{D}(\lambda ){\sigma }_{abs}^{A}(\lambda )d\lambda $$

where *g*_*low*_^*D*^ and *g*_*up*_^*D*^ are the degeneracies of donor (D) states, respectively, from the lower and upper levels involved in the process. *σ*_*em*_^*D*^ and *σ*_*abs*_^*A*^ are emission (donor) and absorption (acceptor) cross section spectra. In this case, the donor and the acceptor are all Ho^3+^ ions, the absorption and emission section can be obtained via Füchtbauer-Ladenburg^44^, Mc-Cumber theory^45^ and showed in Fig. 6(b):

10 $${\sigma }_{em}^{D}(\lambda )=({\lambda }^{5}{A}_{rad}I(\lambda ))/((8\pi c{n}^{2})(\int \lambda I(\lambda )d\lambda ))$$

11 $${\sigma }_{em}^{D}(\lambda )={\sigma }_{abs}^{A}(\lambda )({g}_{low}^{D}/{g}_{up}^{D})\exp [(\varepsilon -h\nu )/kT]$$

where λ is the wavelength, *A*_*rad*_ is the spontaneous radiative transition probability, which can be measured by absorption spectra and Judd-Ofelt parameters theory^46^. *I*(*λ*) is the fluorescence spectra intensity, *n* and *c* represent the refractive index and the speed of light, respectively. *ε* is the net free energy demanded to excite one Ho^3+^ from the ^5^I_7_ to ^5^I_6_ state at the temperature of T.

Basing on Eqs (8–11), *R*_0_ is calculated to be 54.4 Å, and the corresponding critical concentration *N*_0_ is 1.48 × 10^19^cm^−3^.Combining Eqs (6) and (7), the following equation can be expressed as

12 $${\tau }_{m}^{-1}-{\tau }_{rad}^{-1}={W}_{MP}+{W}_{Ho}+\frac{9}{2\pi }\frac{{N}_{Ho}^{3+}(\omega {N}_{OH})}{{\tau }_{rad}{N}_{0}^{2}}$$

Taking the values of *N*_*Ho*_, *N*_0_, and *τ*_*rad*_ into Eq. (12), and then fitting the Eq. (12) to the data shown in Fig. 6(c), we obtained the values of (*W*_*MP*_ + *W*_*Ho*_) and *ω* as 102 ± 0.5 s^−1^, 37.9%, respectively. Thus, the values of *W*_*OH*_ for different OH^−^ concentration can be calculated and showed in Table 3. The maximum quantum efficiency (*η*) of the ^5^I_6_ → ^5^I_7_ transition of Ho^3+^ ions in this fluorotellurite-germanate glass expressed as

13 $$\eta =\frac{{A}_{21}}{{A}_{21}+{A}_{20}+{W}_{Ho}+{W}_{OH}+{W}_{MP}}$$

where A_21_ is the spontaneous transition from levels ^5^I_6_ to^5^I_7_, A_20_ is the spontaneous transition from levels ^5^I_6_ to^5^I_8_, which can be the measured by absorption spectra and Judd-Ofelt parameters theory^46^. All results are listed in Table 3. It can be seen that the quantum efficiency of TG3O sample is 10.09%, which is larger than that of TG3 sample (5.57%). In order to determine the validity of the calculations with the experiments, the quantum efficiency for the (TG1, TG1O), (T1, T1O) and (T3, T3O) samples were evaluated and calculated. The quantum efficiency of (TG1, TG1O) are 5.12% and 10.74%; T1, T1O samples are 6.23% and 10.79%; T3, T3O samples are 6.35% and 10.62%, which are close to the values of TG3 and TG3O samples. Thus, it can be concluded that the calculations are valid. The higher *η* is beneficial for improving corresponding 2.83 μm emission. Therefore, it is concluded that Ho^3+^ activated fluorotellurite-germanate glasses with lower OH^−^ concentrations are promising candidate for 3 μm fiber laser. In addition, the multiphonon processes are another kind of strong nonradiative processes. Thus, the lifetime of the ^5^I_6_ level is predominated by the multiphonon decay except for OH decay. In order to evaluate the contribution of the multiphonen decay compared with the OH decay, the multiphonon decay rate, *W*_*MP*_, can be estimated from the relationship *W*_*MP*_ = *B* * {*exp*[*−α*(*ΔE* − *2hν*_*max*_)]} where B* and α are parameters characteristic of the glass type, i.e., heavy metal glass^47^. For germanate-tellurite glass, B* = 10^6.74^s^−1^ and α = 4.9 × 10^3^ cm. *ΔE* is the energy gap (3600 cm^−1^) between the ^5^I_6_ and ^5^I_7_ levels, which is obtained from the previous measurements of the glasses. *hν*_*max*_ is the maximum glass phonon energy. Thus, the value of *W*_*MP*_ for TG3 glass is 92.63 s^−1^, which is lower than that *W*_*OH*_ (312 s^−1^). From these calculations it is clear that the main decay process for the ^5^I_6_ level is OH decay. Therefore, a higher *η* could be reached by further removing the OH^−^ groups in the future study.

**Table 3:** Measured lifetimes (*τ*_*m*_), calculated lifetime (*τ*_*rad*_) at ^5^I_6_ level of Ho^3+^, spontaneous transitions (A_21_, A_20_) from levels ^5^I_6_ to ^5^I_7_ and ^5^I_8_, energy transfer rate to OH^−^ groups (*W*_*OH*_) and quantum efficiency (*η*) in TG3 and TG3O glasses.

Sample	*τ*_*m*_ (ms)	*τ*_*rad*_ (ms)	A_21_(s^−1^)	A_20_(s^−1^)	W_OH_(s^−1^)	*η*
TG3	0.103	1.45	35.57	108.9	312	5.57%
TG3O	0.218	2.23	35.72	109.4	107	10.09%

## Conclusions

In summary, we systematically studied the spectroscopic and structural properties of Ho^3+^ doped fluorotellurite-germanate glasses activated by Er^3+^, Yb^3+^ ions. Upon excitation at 980 nm, an intense ultra-broad (FWHM = 245 nm) tunable emission at ~3 μm is obtained in Er^3+^/Ho^3+^/Yb^3+^ triply doped fluorotellurite-germanate glass. The glass formation ability and thermal stability of glasses have been improved after introducing GeO_2_ into fluorotellurite glasses. Raman measurement presents the evidences of multiple structural sites and smaller maximum phonon energy (704 cm^−1^) in this fluorotellurite-germanate glass system, which may be in favor of improving the solubility of RE ions and reducing the non-radiative relaxation probability of Ho^3+^ efficiently for enhancing Ho^3+^: 2.8 μm luminescence.

Based on the measured lifetimes and OH^−^ concentrations of the samples, the lifetime quenching mechanism in ^5^I_6_ level of Ho^3+^ ion was also presented and analyzed. The quenching rate to OH^−^ groups decreased from 312 to 107 s^−1^ and quantum efficiency (*η*) increased dramatically from 5.57% to 10.09% by reducing the OH^−^ groups, which indicates that reducing the OH^−^ groups can effectively improve the 3 μm spectroscopic properties of Ho^3+^ doped glasses. All results demonstrate that Ho^3+^ doped fluorotellurite-germanate glass activated by Er^3+^, Yb^3+^ ions is a potential kind of laser glass for efficient 3 μm laser.
